# Genome Characterization of *mcr-1*–Positive *Escherichia coli* Isolated From Pigs With Postweaning Diarrhea in China

**DOI:** 10.3389/fvets.2020.00503

**Published:** 2020-08-25

**Authors:** Liang Guo, Jing Wang, Shenghua Wang, Jinhui Su, Xue Wang, Yaohong Zhu

**Affiliations:** Department of Veterinary Clinical Sciences, College of Veterinary Medicine, China Agricultural University, Beijing, China

**Keywords:** *Escherichia coli*, whole-genome sequencing, multidrug resistance, *mcr-1*, Tn*2*, swine

## Abstract

Diarrheagenic *Escherichia coli* is the causative agent of diarrhea in infants and animals worldwide. Many isolated strains recovered from pigs with postweaning diarrhea are multidrug resistance (MDR), and hybrids of *E. coli* are potentially more virulent, as enterotoxigenic *E. coli* (ETEC)/Shiga-toxigenic *E. coli* (STEC) hybrids. Here, we used whole-genome sequencing to analyze clinical isolates of the five colistin-resistant *E. coli*. The *E. coli* CAU15104, CAU15134, and CAU16060 belonged to ETEC/STEC hybrids, displaying the same serotype O3:H45 and sequence type ST4214. The *E. coli* CAU16175 and CAU16177 belonged to atypical enteropathogenic *E. coli* (aEPEC), display O4:H11 and O103:H2, ST29, and ST20, respectively. The *E. coli* CAU16175 carries six plasmids. An IncHI2-type plasmid, pCAU16175_1, harbors an IS*26*-enriched MDR region, which includes 16 antimicrobial-resistant genes. An IncFII-type plasmid, pCAU16175_3, harbors *mcr-1.1, tet*(M), and *bla*_TEM−1B_, whereas *mcr-1.1* is located within a Tn*2* derivative. Our findings indicate that the ETEC/STEC strains of the O3:H45 serotype as well as the aEPEC strains of the O4:H11 and O103:H2 serotypes are associated with postweaning diarrhea in swine and that some of diarrheagenic *E. coli* contains IS*26*-enriched MDR region and the *mcr-1* gene located within a Tn*2* derivative on IncFII plasmid.

## Introduction

Diarrheagenic *Escherichia coli* (DEC) is a leading cause of infectious diarrhea in humans and animals around the world (1, 2). Diarrheagenic *E. coli* has six well-described pathotypes: enteropathogenic *E. coli* (EPEC), which is subdivided into typical EPEC (tEPEC) and atypical EPEC (aEPEC), enterotoxigenic *E. coli* (ETEC), enteroinvasive *E. coli*, enteroaggregative *E. coli*, diffusely adherent *E. coli*, and enterohemorrhagic *E. coli*, which is subgroup of Shiga-toxigenic *E. coli* (STEC) (3, 4). All of these DECs possess diverse virulence factors, which are encoded by virulence genes and are responsible for their pathogenicity (5). Enterotoxigenic *E. coli* strains typically produce one or two toxins, heat-labile enterotoxin (LT) encoded by *ltc*, and heat-stable enterotoxin (ST) encoded by *st* (6). In pigs, STEC strains are characterized by producing the Shiga-like toxin variant Stx2e encoded by *stx2e* (7). Enteropathogenic *E. coli* strains are defined as forming the attaching and effacing (A/E) lesions mediated by genes located on the locus of enterocyte effacement (LEE) pathogenicity island (including *eae*) in the intestinal epithelium but not produce Shiga-like toxin (Stx) (8). Moreover, *bfpA*, which encodes the major subunit of bundle forming pili, is used to subdivide EPEC into tEPEC and aEPEC. Hence, aEPEC strains are defined by *eae*^+^, *bfpA*^−^, and *stx*^−^ (9). Three pathotypes (ETEC, STEC, EPEC) are major DEC causing postweaning diarrhea (PWD) in pigs, and many hybrids of *E. coli* (ETEC/STEC, ETEC/STEC/EPEC) are present during PWD (6, 10). The recent data from The Global Enteric Multicenter Study showed that tEPEC and ETEC associated with a higher risk of fatal outcomes in children younger than 24 months with moderate to severe diarrhea (11). Atypical EPEC has outbreaks linked to diarrhea in children (12).

The occurrence of antimicrobial resistance and prevalence of multidrug resistance (MDR) Gram-negative Enterobacteriaceae has been increasing worldwide between humans and animals (13). In the cases of PWD, antimicrobial treatment has been widely used and caused severe drug resistance in DEC (1, 6, 14). In our previous study, among the 171 *E. coli* isolates, 94.15% of the strains were MDR, with antimicrobial resistance rates ranging from 2.34% for meropenem to 90.05% for nalidixic acid (6). Among the ETEC strains that cause PWD, resistance to apramycin, neomycin, trimethoprim sulfonamide, and colistin has been increasingly observed (1). Multidrug resistance strains may spread from animals to humans, causing antibiotics ineffective and increasing mortality and morbidity in developing countries (15).

Colistin as the last resort treatment against MDR bacterial infections may have been challenged by the mobile colistin-resistant gene (*mcr-1*), which has received widespread attention in different species of Enterobacteriaceae found in animals and humans around the world since it was first reported (16–19). So far, nine allelic variants of *mcr-1* (*mcr-2* to *mcr-10*) have been detected (20, 21). In addition, they have large number of variants, such as *mcr-1* (*mcr-1.1* to *mcr-1.22*), *mcr-2* (*mcr-2.1* to *mcr-2.3*), *mcr-3* (*mcr-3.1* to *mcr-3.30*), *mcr-4* (*mcr-4.1* to *mcr-4.6*), *mcr-5* (*mcr-5.1* to *mcr-5.4*), and *mcr-8* (*mcr-8.1* and *mcr-8.2*) (22). Our previous study found that the resistance rate to colistin was 20.47% in 171 *E. coli* isolates (6), and a recent article reported that direct sample testing rates of *mcr-1* were higher than the rates of *mcr-1*–positive *E. coli* (64.6 vs. 49.2%) (23).

Insertion sequences (ISs) and transposons (Tns), arguably most numerous autonomous transposable elements, are crucial to shape their host genomes, particularly important in the bacterial antimicrobial resistance (24). IS*26* and Tn*2*, which is a member of the IS*6* family and Tn*3* family, respectively, play a key role in the dissemination of antimicrobial-resistant genes in Gram-negative bacteria (25, 26). IS*26* is often existed in MDR Gram-negative bacteria, which usually carry large regions containing several antimicrobial-resistant genes that are flanked by and interspersed with copies of IS*26* (25). Tn*2* is the most abundant in commensal *E. coli*. (27). IS*Swi1*-m2 in pNJST258C2 is a derivative of Tn*2* that includes IS*26* compared with Tn*2* (28). IS*Apl1*, which is an IS initially identified in *Actinobacillus pleuropneumoniae*, is a member of the IS*30* family and is considered to be an essential element in the mobilization of *mcr-1* (29, 30).

In the present study, we used whole-genome sequencing (WGS) to analyze virulence and resistant genes of the five clinical colistin-resistant *E. coli* isolates recovered from pigs with PWD and to characterize the complete sequence of a Tn*2* derivative carrying *mcr-1.1* on IncFII plasmid, pCAU16175_3, from a swine aEPEC isolate.

## Materials and Methods

### Bacterial Isolation and Identification

A total of 455 *E. coli* strains were obtained from feces samples or small intestinal content from pigs with diarrhea in China between 2014 and 2016 and identified with polymerase chain reaction (PCR) amplification of the *uspA* gene (6). Of these, 171 *E. coli* isolates were further screened by antimicrobial susceptibility testing, and the resistance rate of colistin was 20.47% (6). From these 35 colistin-resistant *E. coli*, we observed ST4214 was a major clone (6/35, 17.14%) based on the multilocus sequence typing and caused severe damage to IPEC-J2 cells (6). Then, ST4214 *E. coli* CAU15014, CAU15134, and CAU16060 were selected from different swine farms (one isolate per farm), and two MDR aEPEC isolates (*E. coli* CAU16175 and CAU16177) were randomly chosen for WGS.

### Antimicrobial Susceptibility Testing

The susceptibility of the five isolates to 18 antimicrobials were tested by determining the minimum inhibitory concentration (MIC) using the US Clinical and Laboratory Standards Institute (CLSI) broth micro method (31). The results of MIC for ampicillin (AMP), co-amoxiclav (AMC), cefazolin (CZ), kanamycin (KAN), gentamicin (GEN), amikacin (AMK), tetracycline (TE), trimethoprim-sulfamethoxazole (SXT), ciprofloxacin (CIP), nalidixic acid (NAL), chloramphenicol (CHL), and nitrofurantoin (NIT) were interpreted according to guidelines of CLSI 2016 M100-S26 (31). The results of MIC for ceftiofur (EFT), enrofloxacin (ENR), and florfenicol (FFC) were interpreted according to CLSI VET01-A4 (32). In addition, the results with MIC values were defined resistant: streptomycin (STR) ≥64 μg/mL (33), olaquindox (OLA) ≥64 μg/mL (34), and polymyxin B (PB) >2 μg/mL (35). *Escherichia coli* ATCC 25922 was used as the quality control. According to the MIC determined for each antimicrobial, the isolates were defined as “susceptible,” “intermediate,” or “resistant.”

### Whole-Genome Sequencing

Bacterial isolates were recultured from stock; DNA was extracted using a TIANamp Bacteria DNA kit (Tiangen Biotech Inc., Beijing, China). We used WGS by the Illumina Hiseq platform to get draft genome of the five *E. coli* isolates and further used WGS by the Oxford Nanopore Technologies MinION platform to get complete genome of the *E. coli* CAU16175.

The complete genome assembly was constructed from the two sequence data sets using Unicycler (Shanghai Majorbio Bio-pharm Technology Co., Ltd., Shanghai, China). The serotype, multilocus sequence type, plasmid type, antimicrobial-resistant gene, and virulence gene detection were performed using the Center for Genomic Epidemiology server (https://cge.cbs.dtu.dk). Insertion sequence typing was carried out using the ISFinder database (https://www-is.biotoul.fr/). The complete genome sequences were initially annotated with Rapid Annotation using the Subsystem Technology server (http://rast.nmpdr.org) and curated manually using the BLAST server (https://blast.ncbi.nlm.nih.gov/Blast). The obtained plasmid sequences were aligned with homologous plasmid sequences from NCBI using the BRIG tool (36).

### Conjugation Assay

The conjugation experiment was carried out using the *E. coli* CAU16175 as the donor and the *E. coli* J53 (resistant to sodium azide) as the recipient. The transconjugant was screened on BHI agar plates containing sodium azide (150 mg/L) and colistin (4 mg/L). The presence of *mcr-1* in the transconjugant was confirmed by PCR, and the MICs of antimicrobial agents for the transconjugant were determined using the agar dilution method. The conjugation experiment was repeated three times, and the conjugation frequencies were calculated as the number of transconjugants per recipient. The transconjugant strains were distinguished from donor occurring natural mutants of sodium azide–resistant *E. coli* by verifying *eae*, which is marker gene for *E. coli* CAU16175 donor.

### GenBank Accession Number

The draft genome sequences of the *E. coli* CAU15104, CAU15134, CAU16060, and CAU16177 isolates have been deposited at GenBank under SRA accession no. SRR10828049, SRR10828048, SRR10828047, and SRR10828046, respectively. The complete genome sequences of the *E. coli* CAU16175 have been deposited at GenBank under SRA accession no. SRR10813965.

## Results

### Antimicrobial Susceptibility Testing of the Five *E. coli* Isolates

Antimicrobial susceptibility testing of the five isolates showed that they were MDR exhibiting resistant to at least three different classes of antimicrobials (Table 1). All the isolates showed MDR to KAN, STR, TE, SXT, NAL, and PB.

**Table 1:** Antimicrobial susceptibility profile of the five *E. coli* isolates.

**Strain**	**Antibiotics**																	
	**AMP**	**AMC**	**EFT**	**CZ**	**KAN**	**GEN**	**STR**	**AMK**	**TE**	**SXT**	**CIP**	**ENR**	**NAL**	**CHL**	**FFC**	**NIT**	**OLA**	**PB**
CAU15104	S	R	R	R	R	S	R	I	R	R	R	R	R	R	S	S	I	R
CAU15134	R	S	S	S	R	S	R	S	R	R	S	S	R	S	I	I	S	R
CAU16060	R	R	I	S	R	S	R	S	R	R	I	R	R	R	S	R	I	R
CAU16175	R	R	R	R	R	R	R	S	R	R	R	R	R	R	R	S	I	R
CAU16177	S	R	R	S	R	R	R	S	R	R	S	I	R	R	S	R	I	R

*AMP, ampicillin; AMC, co-amoxiclav; EFT, ceftiofur; CZ, cefazolin; KAN, kanamycin; GEN, gentamicin; STR, streptomycin; AMK, amikacin; TE, tetracycline; SXT, trimethoprim-sulfamethoxazole; CIP, ciprofloxacin; ENR, enrofloxacin; NAL, nalidixic acid; CHL, chloramphenicol; FFC, florfenicol; NIT, nitrofurantoin; OLA, olaquindox; PB, polymyxin B*.

### Characterization of the *E. coli* Isolates From the Draft Genome Sequences

The clinical isolates of the *E. coli* CAU15104, CAU15134, and CAU16060 belonged to ETEC/STEC (*ltcA*^+^, *stb*^+^, and *stx*^+^) hybrid strains, display the same serotype O3:H45 and sequence type ST4214, which are identical sequence type with the strains swine19 (LVMV00000000), swine22 (LVMY00000000), swine54 (LVMV00000000), and swine67 (LVOR00000000) from the NCBI database, all carrying *fedA, fedF, iha, stb, ltcA, sepA, stx2A*, and *stx2B* virulence genes and antimicrobial-resistant genes conferring resistant to KAN (*aph(3*′*)-Ia*), STR (*aadA2, strA*, and *strB*), TE [*tet*(A) and *tet*(M)], SXT (*dfrA12, sul1, sul2*, and *sul3*) and PB (*mcr-1.1*) (Tables 1, 2). Besides, the *E. coli* CAU15104, CAU15134, and CAU16060 carry *cmlA1* and *floR*, which are associated to phenicol antibiotic (Table 2). The *E. coli* CAU15104 and CAU15134 carry *qnrS2* (associated to quinolone antibiotic) and *aac(6*′*)Ib-cr* (associated to quinolone and aminoglycoside antibiotic). The *E. coli* CAU15134 and CAU16060 carry *dfrA1* (associated to SXT), *mcr-3.1* (associated to PB), *oqxA*, and *oqxB*, which are efflux pump conferring antibiotic resistance, and exhibit resistant to AMP associated to *bla*_TEM−1B_.

**Table 2:** Overview of the molecular typing, antimicrobial resistance genes and virulence genes of the five *E. coli* isolates from the whole-genome sequencing result by the Illumina Hiseq platform (CAU16175 Illumina/Nanopore) and four ST4214 *E. coli* strains from the NCBI database.

**Strain**	**Collection date**	**Collection province**	**Serotype**	**MLST**	**Plasmid type**	**Antimicrobial resistance gene**	**Virulence gene**	**GenBank accession no**.
CAU15104	2015	Shandong	O3:H45	ST4214	IncF, IncI1	*aac(6′)Ib-cr, aadA1, aadA2, aph(3′)-Ia, bla*_CMY−2_, *cmlA1, dfrA12, erm*(B), *floR, mcr-1.1, mph*(A), *qnrS2, strA, strB, sul1, sul2, sul3, tet*(A), *tet*(M)	*fedA, fedF, iha, ltcA, sepA, stb, stx2A, stx2B*	WVUR00000000
CAU15134	2015	Liaoning	O3:H45	ST4214	IncF, IncI1, IncHI2	*aac(6′)Ib-cr, aadA2, aph(3′)-Ia, bla*_TEM−1B_, *cmlA1, dfrA1, dfrA12, floR, mcr-1.1, mcr-3.1, oqxA, oqxB, qnrS2, strA, strB, sul1, sul2, sul3, tet*(A), *tet*(M)	*astA, fedA, fedF, gad, iha, ltcA, sepA, sta1, stb, stx2A, stx2B*	WVUQ00000000
CAU16060	2016	Shandong	O3:H45	ST4214	IncF, IncHI2	*aadA2, aph(3′)-Ia, bla*_TEM−1B_, *cmlA1, dfrA1, dfrA12, floR, mcr-1.1, mcr-3.1, oqxA, oqxB, strA, strB, sul1, sul2, sul3, tet*(A), *tet*(M)	*fedA, fedF, gad, iha, ltcA, sepA, sta1, stb, stx2A, stx2B*	WVUP00000000
CAU16175	2016	Hunan	O4:H11	ST29	IncHI2, IncFIB, IncFII	*aac(3)-IVa, aac(6′)Ib-cr, aadA1, aadA2, aph(3′)-Ia, aph(4)-Ia, ARR-3, bla*_OXA−1_, *bla*_TEM−1B_, *catB3, cmlA1, dfrA12, floR, mcr-1.1, sul1, sul2, sul3, tet*(A), *tet*(M)	*astA, cif, eae, efa1, espA, espB, espJ, gad, iha, iss, katP, lpfA, nleB, nleC, perA, toxB, tir*	CP047378-CP047384
CAU16177	2016	Hunan	O103:H2	ST20	IncF, IncHI2	*aac(3)-IVa, aac(6′)Ib-cr, aph(3′)-Ia, aph(4)-Ia, ARR-3, bla*_OXA−1_, *bla*_OXA−10_, *catB3, cmlA1, dfrA12, dfrA14, floR, mcr-1.1, mcr-3.1, oqxA, oqxB, qnrS1, strA, strB, sul1, sul2, sul3, tet*(A)	*cif, eae, espA, espB, espF, espJ, gad, iss, nleA, nleB, tir*	WVUO00000000
swine19	2014	Jiangsu	O130:H45	ST4214	IncA/C	*aadA2, strA, strB, mcr-1.1, sul3, sul1, tet*(A), *cmlA1, bla*_TEM−1B_, *oqxB, oqxA*	*astA, fedA, fedF, iha, sta1, stb, stx2A, stx2B*	LVMV00000000
swine22	2014	Jiangsu	O130:H45	ST4214	IncA/C	*aadA2, strA, strB, mcr-1.1, sul3, sul1, tet*(A), *cmlA1, bla*_TEM−1B_, *oqxB, oqxA*	*astA, fedA, fedF, iha, sta1, stb, stx2A, stx2B*	LVMY00000000
swine54	2012	Jiangsu	O3:H45	ST4214	IncA/C	*aph(3')-Ia, strA, strB, aadA2, mcr-1.1, sul3, sul2, sul1, dfrA12, tet*(A), *tet*(M), *cmlA1, floR, bla*_TEM−1B_, *oqxA, oqxB*	*astA, fedA, fedF, gad, ltcA, iha, sepA, sta1, stb, stx2A, stx2B*	LVOE00000000
swine67	2012	Jiangsu	O3:H45	ST4214	IncA/C	*aadA2, aph(3')-Ia, strA, strB, sul3, sul2, sul1, oqxB, oqxA, qnrS2, dfrA12, tet*(A), *tet*(M), *mcr-1.1, cmlA1, floR, bla*_TEM−1B_, *aac(6')Ib-cr*	*astA, fedA, fedF, ltcA, iha, stb, sepA, stx2A, stx2B*	LVOR00000000

The clinical isolates of the *E. coli* CAU16175 and CAU16177, belonged to aEPEC (*eae*^+^, *bfpA*^−^, and *stx*^−^), display different serotype and sequence type, O4:H11 and O103:H2, ST29 and ST20, respectively. They both carry *cif*, *eae, espA, espB, espJ, gad, iss, nleB*, and *tir* virulence genes and antimicrobial-resistant genes conferring resistance to beta-lactam antibiotic (*bla*_OXA−1_), KAN (*aph(3*′*)-Ia*), GEN (*aac(3)-IVa*), STR (*aadA1* or *aadA2* or *strA* or *strB*), TE [*tet*(A) and/or *tet*(M)], SXT (*dfrA12, sul1, sul2, sul3*, and/or *dfrA14*), and PB (*mcr-1.1* and/or *mcr-3.1*) (Tables 1, 2). In addition, the *E. coli* CAU16175 and CAU16177 both carry *aac(6*′*)Ib-cr* (associated to quinolone and aminoglycoside antibiotic), *aph(4)-Ia* (associated to aminoglycoside antibiotic), *ARR-3* (associated to rifamycin antibiotic), *catB3, cmlA1*, and *floR* (associated to phenicol antibiotic) (Table 2). Interestingly, we observed that the *mcr-1.1* gene and the *bla*_TEM−1B_ gene existed in the same scaffold of the *E. coli* CAU16175 draft genome. Besides, partial sequences of this scaffold including *mcr-1.1* and *bla*_TEM−1B_ are highly homologous with Tn*2* (KT002541).

### Complete Genome of the *E. coli* CAU16175

The 6.07-Mb complete genome of *E. coli* CAU16175 has a total GC content of 50.47% with a single chromosome and six plasmids. The 5.62 Mb chromosome has a GC content of 50.66%. The six plasmids sequences of *E. coli* CAU16175 range in length from 6.99 to 190.22 kb with a GC content from 47.10 to 51.35% (Table 3). The chromosome harbors LEE T3SS effectors, non-LEE T3SS effectors, and *mdf* (A) antimicrobial-resistant gene. The pCAU16175_1 (IncHI2 type) harbors an IS*26*-enriched MDR region, which includes *bla*_OXA−1_ and 15 additional antimicrobial-resistant genes. The pCAU16175_2 (IncFIB type) harbors *katP, sepA*, and *perA* virulence genes. The pCAU16175_3 (IncFII type) harbors *mcr-1.1, tet*(M), and *bla*_TEM−1B_ antimicrobial-resistant genes. The pCAU16175_5 harbors *celb* virulence gene (Table 3).

**Table 3:** Genome summary for ST29 aEPEC isolate CAU16175.

	**Sequence length (bp)**	**GC%**	**Plasmid rep type(s)**	**Antimicrobial resistance gene**	**Virulence region or gene**	**GenBank accession no**.
Chromosome	5615389	50.66	NA	*mdf*(A)	LEE, non-LEE T3SS effectors	CP047378
pCAU16175_1	190219	47.10	IncHI2	*aac(6')-Ib-cr, sul1, sul2, sul3, bla*_OXA−1_, *tet*(A), *ARR-3, aac(3)-IVa, aadA1, aadA2b, aph(3')-Ia, aph(4)-Ia, catB3, cmlA1, floR, dfrA12*		CP047379
pCAU16175_2	161176	47.91	IncFIB (AP001918)		*katP, sepA, perA*	CP047380
pCAU16175_3	76633	51.35	IncFII (29)	*tet*(M), *mcr-1.1, bla*_TEM−1B_		CP047381
pCAU16175_4	10675	49.23	—			CP047382
pCAU16175_5	7098	50.52	—		*celb*	CP047383
pCAU16175_6	6988	47.58	—			CP047384

### Conjugation Assay

The susceptibility testing of polymyxin B and E showed that the MIC values for the transconjugants (J53-*mcr-1*) increased to 8 and 16 mg/L, respectively (Table 4). The conjugation frequency was 2.1 × 10^−8^ transconjugants per recipient.

**Table 4:** Minimum inhibitory concentration (mg/L) for CAU16175, J53, and J53-*mcr-1*.

	**CAU16175**	**J53**	**J53-*mcr-1***
TE	32	1	1
GEN	>32	0.5	0.5
AMK	16	16	2
KAN	>128	8	2
AMP	>64	4	4
CFZ	>16	1	1
CIP	>16	<0.125	<0.125
CHL	32	1	2
RFP	<1	<1	<1
PB	**4**	**1**	**8**
PE	**16**	**2**	**16**
STR	>256	32	2
NaN_3_	<75	300	300
EFT	16	<1	<1
FFC	32	8	4
AMC	>16	4	4

*TE, tetracycline; GEN, gentamicin; AMK, amikacin; KAN, kanamycin; AMP, ampicillin; CFZ, cefazolin; CIP, ciprofloxacin; CHL, chloramphenicol; RFP, rifapentine; PB, polymyxin B; PE, polymyxin E; STR, streptomycin; EFT, ceftiofur; FFC, florfenicol; AMC, co-amoxiclav. Highlight the colistin resistance was transferred to J53*.

### Genetic Characterization of the pCAU16175_1 Harboring the MDR Region

**Figure 1:**
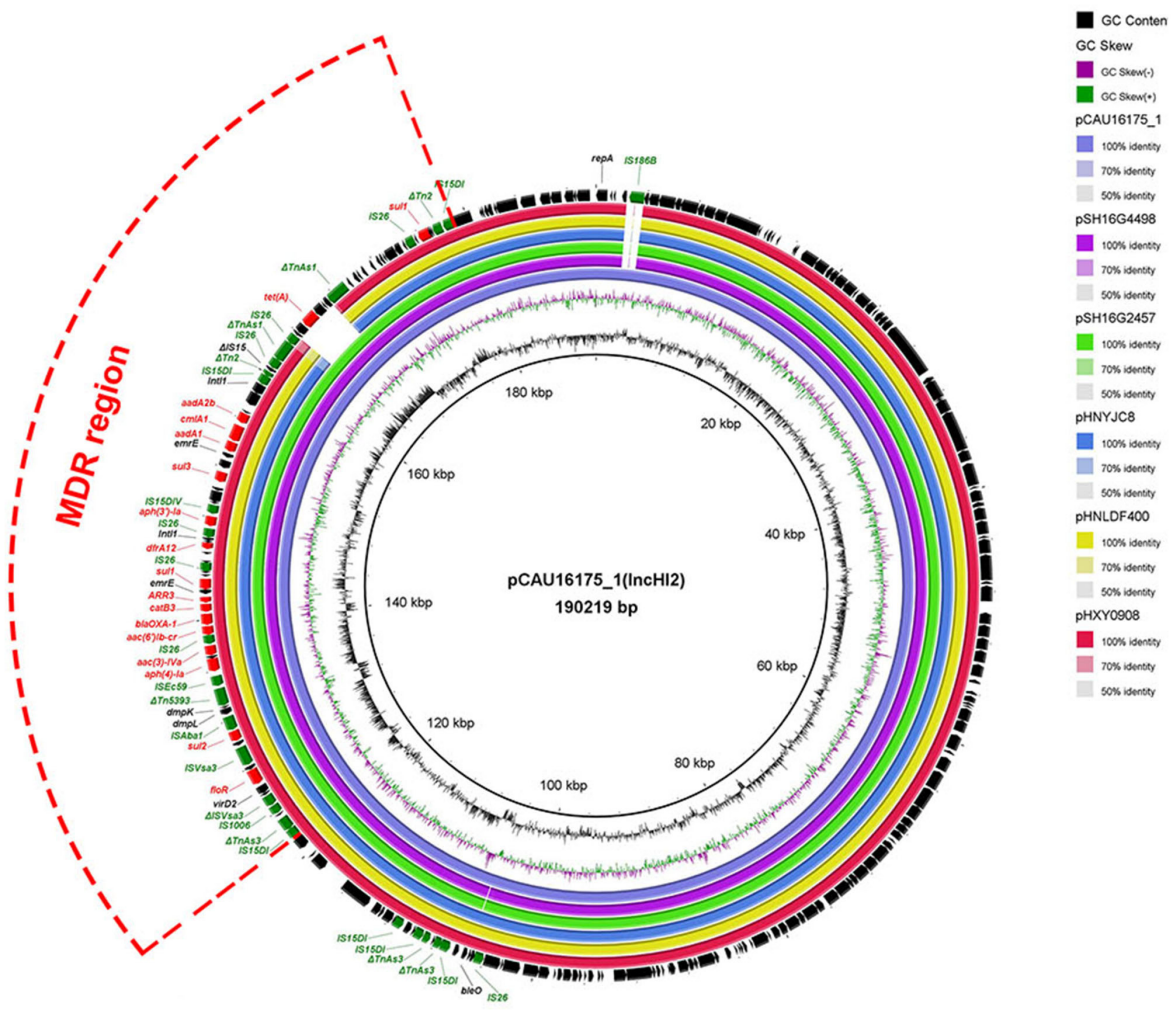
Comparative analysis of MDR-carrying plasmids pCAU16175_1, pSH16G4498, pSH16G2457, pHNYJC8, pHNLDF400, and pHXY0908 using the BLAST Ring Image Generator. The concentric rings show similarity compared the pCAU16175_1 as reference plasmid in inner ring with the other plasmids in outer rings. The varying color levels indicate a BLAST result with matched degree of shared regions, as shown to the right of the ring. The outer circle with red arrows and green arrows denotes antimicrobial resistance gene and transposable elements, respectively. Detailed information of the complete sequences of IncHI2 plasmids is described in Supplementary Table 1.

The 190.22-kb plasmid pCAU16175_1 was blasted against the GenBank nucleotide collection (nr/nt) database. An overall nucleotide sequence identity (99.66–99.86%) with query coverages of 90–99% to pSH16G4498 (MH522423), pSH16G2457 (MH522421), pHNYJC8 (KY019259), pHNLDF400 (KY019258), pHXY0908 (KM877269), and other 25 IncHI2-type plasmids were observed (Supplementary Table 1). We chose the five most similar plasmids for comparison analysis; these plasmids have MDR regions and many IS*6* family insert sequences (IS*26*/IS*15DI*/IS*15DIV*/IS*1006*/IS*Ec59*) (Figure 1). The pCAU16175_1 shows almost exactly the same sequence in MDR region with pSH16G4498 and pSH16G2457, which both existed in *Salmonella typhimurium* recovered from humans in China (Supplementary Table 1). In the pCAU16175_1, the MDR region locates at nucleotide location 121,521–178,916 (56,625 bp). The structure of the MDR region comprises twelve IS*6* family insert sequence [including six copies IS*26*, three copies IS*15DI* (3 bp differ with IS*26*), one copy IS*15DIV* (1 bp differ with IS*26*), one copy IS*1006*, and one copy IS*Ec59*] that flank containing different antimicrobial-resistant genes. The MDR region contains *floR, sul2, aph(4)-Ia, aac(3)-IVa, aac(6')-Ib-cr, bla*_OXA−1_, *catB3, ARR-3, sul1* (two copies), *dfrA12, aph(3')-Ia, sul3, aadA1, cmlA1, aadA2b*, and *tet*(A). The MDR region is also interspersed with a number of different mobile elements including ΔTn*As3*, ΔIS*Vsa3*, IS*Vsa3*, IS*Aba1*, ΔTn*5393*, ΔTn*2*, ΔIS*15*, and ΔTn*As1*.

### Genetic Characterization of the pCAU16175_3 Harboring the *mcr-1.1* Gene

**Figure 2:**
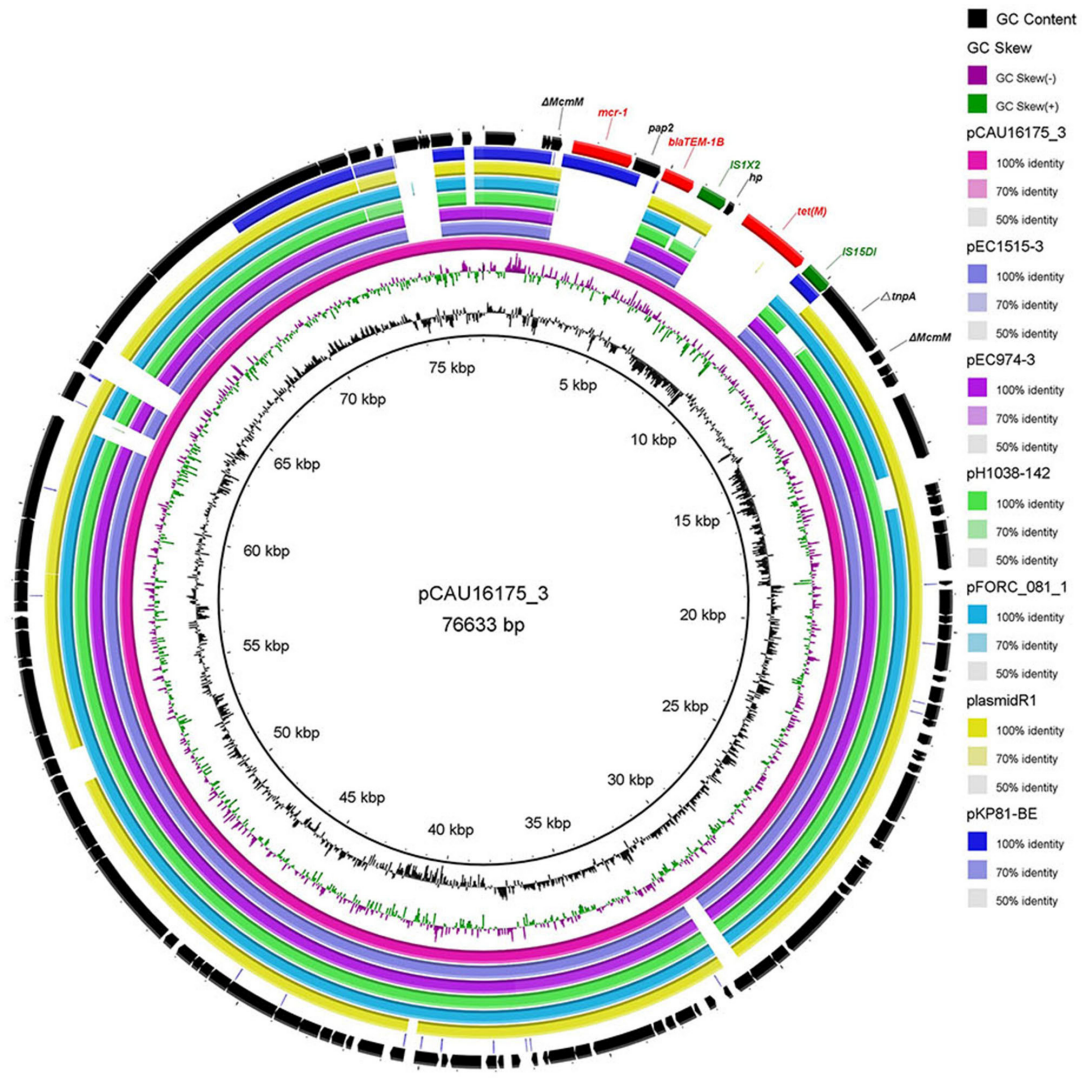
Comparative analysis of seven IncFII-type plasmids pCAU16175_3, pEC1515-3, pEC974-3, pH1038-142, pFORC_081_1, plasmid R1, and pKP81-BE (carrying *mcr-1*) using the BLAST Ring Image Generator. The concentric rings show similarity compared the pCAU16175_3 as reference plasmid in inner ring with the other plasmids in the outer rings. The varying color levels indicate a BLAST result with matched degree of shared regions, as shown to the right of the ring. The outer circle with red arrows and green arrows denotes antimicrobial resistance gene and transposable elements, respectively. Detailed information of the complete sequences of IncFII plasmids is described in Supplementary Table 2.

The 76.63-kb plasmid pCAU16175_3 was blasted against the GenBank Nucleotide collection (nr/nt) database. An overall nucleotide sequence identity (94.46–97.88%) with query coverages of 80–89% to pEC1515-3 (CP021847), pEC974-3 (CP021843), pH1038-142 (KJ484634), pFORC_081_1 (CP029058), plasmid R1 (KY749247), and other 27 IncFII-type plasmids were observed (Supplementary Table 2). We chose five most similar IncFII-type plasmids and reported IncFII-type plasmid-*mcr-1* pKP81-BE (KU994859) for comparison analysis (Figure 2). Although the pCAU16175_3 demonstrates highly sequence homology with the other five IncFII-type plasmids, the *mcr-1.1* gene only exists in pCAU16175_3 and pKP81-BE. Besides the *mcr-1.1* gene, the pCAU16175_3 also carries the antimicrobial-resistant gene *bla*_TEM−1B_ and *tet*(M), which are close to the *mcr-1.1* gene.

**Figure 3:**
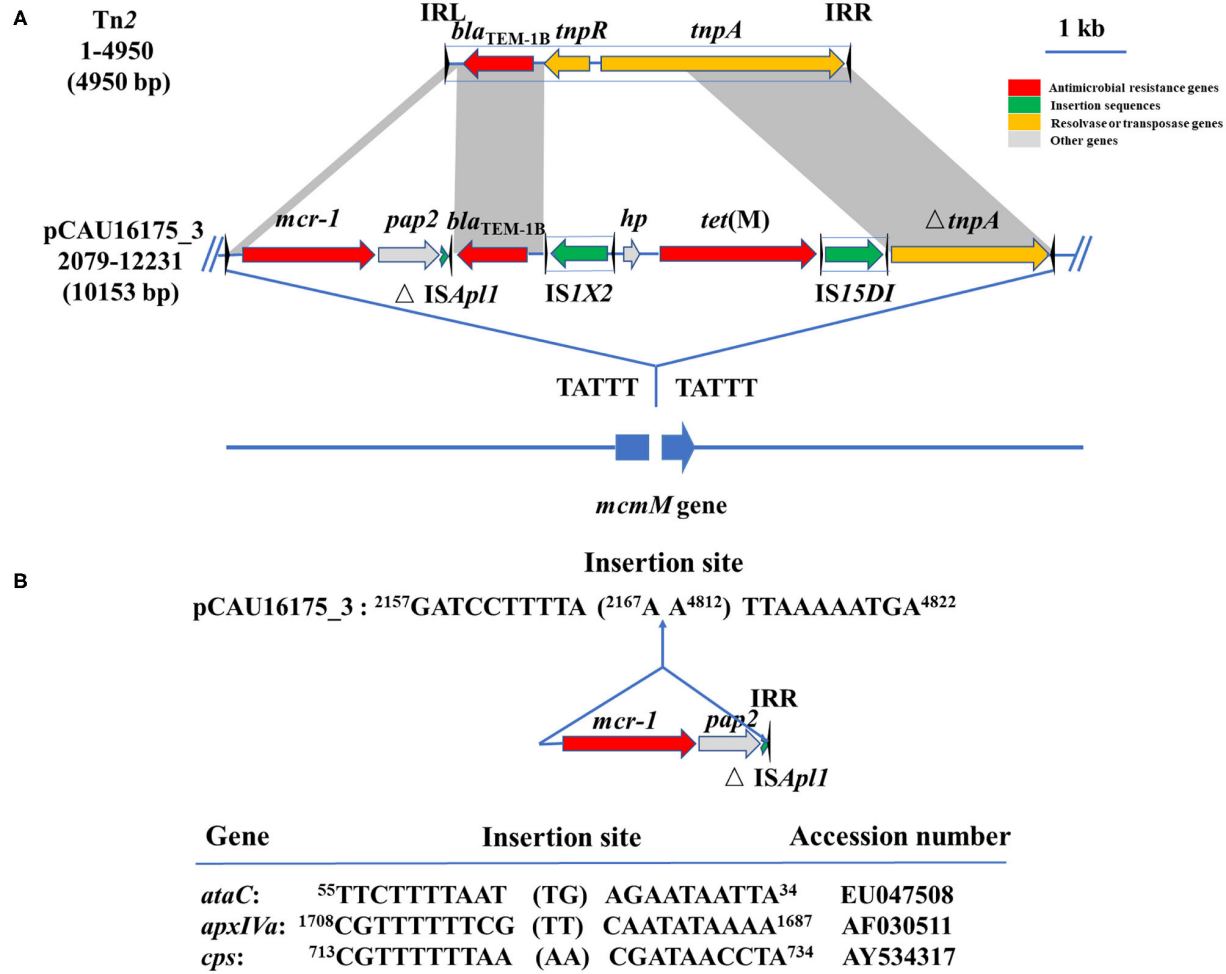
Genetic features of pCAU16175_3 (nucleotide location 2079-12231). **(A)** Linear sequence comparison of pCAU16175_3 (nucleotide location 2079-12231) with Tn*2*. Arrows indicate the positions and directions of the genes, Δ indicates a truncated gene. Regions with >99% homology are indicated by gray shading. Inverted repeat nucleotide sequences (IRL: left IR; IRR: right IR) of IS are marked by triangles. Antimicrobial resistance genes, insertion sequences, resolvase or transposase genes, and other genes are indicated by red, green, khaki, and gray, respectively. **(B)** DNA sequence of insertion sites of *mcr-1*-*pap2*-ΔIS*Apl1* in pCAU16175_3 and IS*Apl1* in *ataC, apxIVA*, and *cps* gene (30). Shown sequences represent 10 base pairs upstream and downstream of insertion site, and 2-base-pair direct repeats in brackets. Numbers represent positions in the indicated sequences deposited in GenBank.

Noticeably, the nucleotide location of the pCAU16175_3 from 2,079 to 12,231 has high homology with the Tn*2* derivative (Figure 3A). This structure is a unique identification compared with the NCBI database and was confirmed by conventional PCR (Supplementary Figure 1A). The genetic content of the structure analysis showed that the structure is located within *mcmM* (encoded microcin M). A five-nucleotide direct duplication (TATTT) was identified flanking the structure. Unlike the Tn*2*, this structure harbors *mcr-1*–*pap2*-ΔIS*Apl1* and IS*1X2*-*hp*-*tet*(M)-IS*15DI*, deletes the resolvase (*tnpR*), and truncates the transposase (*tnpA*). Besides, the striking features of the insertion sites of IS*Apl1* were found, which includes high AT content and 2-bp direct repeats (DRs) (AA) (Figure 3B). From the result of conjugation experiment and PCR, the whole structure gene did not occur transposition (Table 4, Supplementary Figure 1B). The *mcr-1* gene was transferred to J53 without the *pap2* gene, whereas colistin resistance was transferred to J53. IS*15DI* was also transferred to J53.

## Discussion

Postweaning diarrhea caused by DEC is an economically important disease for the swine industry around the world. In our previous study, we collected 455 *E. coli* isolates recovered from feces samples or small intestinal content from pigs with diarrhea in China between 2014 and 2016 to know the *E. coli* pathotype and the antimicrobial susceptibility of the isolates (6). Most of the isolates belonged to ETEC, followed by aEPEC, which is similar to the report in Spain; that is, most cases of PWD are significantly associated with ETEC (67%) and aEPEC (21.7%) (37). Our previous study showed that 95.91% of 171 *E. coli* isolates were MDR exhibiting resistance to at least three different classes of antimicrobials, and 20.47% *E. coli* isolates were resistant to colistin. It has been revealed that MDR isolates that existed in swine industry are associated with the widespread use of antibiotics (38, 39). Overuse of colistin is considered to contribute to the emergence and spread of *mcr-1* (40). The 36 *mcr-1*–positive *E. coli* isolates recovered from pigs with PWD showed MDR (29). In this study, we used WGS to analyze the five MDR (including colistin resistance) of *E. coli* isolates from pigs with PWD and found that they demonstrate different serotype and sequence type and carry different antimicrobial-resistant genes and virulence genes.

The clinical isolates of the *E. coli* CAU15104, CAU15134, and CAU16060 belonged to ETEC/STEC, displaying the same serotype O3:H45 and sequence type ST4214. As far as we know, there are no reports about this clone strains, and only four have WGS of these clone strains. The *E. coli* swine19, swine22, swine54, and swine67 also belong to ETEC/STEC and display the same sequence type ST4214. The *E. coli* swine19 and swine22 display the same serotype O130:H45. The *E. coli* swine54 and swine67 display the same serotype O3:H45, similar to our collected the *E. coli* CAU15104, CAU15134, and CAU16060. Five ETEC/STEC isolates recovered from pigs with enteric colibacillosis in Spain display the serotype O141:H4 and sequence type ST10 (37). In the present study, all of the ST4214 ETEC/STEC we collected were MDR (at least three different classes of antimicrobials) and showed MDR to PB, TE, and NAL, carried 17–19 antimicrobial-resistant genes and 8–11 virulence genes. Besides, all the ST4214 ETEC/STEC harbored aminoglycoside-resistant genes (*aadA2* and *aph(3*′*)-Ia*), phenicol-resistant genes (*cmlA1* and *floR*), tetracycline-resistant genes [*tet*(A) and *tet*(M)], trimethoprim-resistant gene (*dfrA12*), sulfonamide-resistant genes (*sul1, sul2*, and *sul3*), streptomycin-resistant genes (*strA* and *strB*), and colistin-resistant genes (*mcr-1.1*). The ST4214 ETEC/STEC also harbored fimbrial adhesin genes (*fedA* and *fedF*), adherence gene (*iha*), heat-labile enterotoxin (LT) gene (*ltcA*), heat-stabile enterotoxin (ST) gene (*stb*), *Shigella* extracellular protein gene (*sepA*), and Shiga toxin 2 variant e genes (*stx2A* and *stx2B*). We observed the ST4214 strains were recovered from different provinces (Shandong, Jiangsu and Liaoning) and years (2012, 2014, 2015, and 2016) in China, which indicated this clone strains have spread in swine and should be brought to our attention.

In addition, the clinical isolates of the *E. coli* CAU16175 and CAU16177 belonged to aEPEC, displaying different serotype and sequence type, O4:H11 and O103:H2, ST29 and ST20, respectively. For aEPEC, serotypes O45 and O123 are frequently occurring in diarrheagenic pigs (41, 42). The *E. coli* CAU16175 and CAU16177 also showed MDR to AMC, KAN, PB, TE, NAL, and CHL and carried 19/23 antimicrobial-resistant genes and 17/11 virulence genes. They both harbored beta-lactamase-resistant genes (*bla*_OXA−1_), aminoglycoside-resistant genes (*aac(3)-IVa, aac(6*′*)Ib-cr, aph(3*′*)-Ia* and *aph(4)-Ia*), phenicol-resistant genes (*cmlA1, catB3*, and *floR*), tetracycline-resistant genes [*tet*(A)], trimethoprim-resistant gene (*dfrA12*), sulfonamide-resistant genes (*sul1, sul2*, and *sul3*), rifampicin-resistant gene (*ARR-3*), and colistin-resistant genes (*mcr-1.1*). Interestingly, *E. coli* CAU16177 also harbored *mcr-3.1*. Four *mcr-1*– and *mcr-3*-positive *E. coli* have been reported, and three *E. coli* isolates were recovered from pigs (23). The *E. coli* CAU16175 and CAU16177 both harbored type III secretion system-associated virulence genes (*cif*, *espA, espB*, and *espJ*), non–LEE-encoded effector gene (*nleB*), glutamate decarboxylase gene (*gad*), increased serum survival gene (*iss*), a marker gene for EPEC, intimin gene (*eae*), and translocated intimin receptor gene (*tir)*.

The *E. coli* CAU16175 carries IS*26*-enriched MDR region in pCAU16175_1 (IncHI2 type). The IncHI2-type plasmids have been discovered as genetic elements mediating the transmission of MDR genes (16). IS*26*-flanked Tns play an increasingly critical role in the mobilization and development of antimicrobial-resistant genes (43–45). So far, there are 51 Tns related with IS*26* from the Tn registry website. In an individual *E. coli* strain heterogeneous resistance-encoding plasmid, polymorphic MDR regions driven by IS*26*-flanked Tns have been detected (44). From the BLAST analysis, we found that there were abundant IS*26*-enriched MDR regions in *Salmonella* and *E. coli*, which were mostly isolated from humans and animals. The results showed that the plasmid carrying IS*26*-enriched MDR region has been widely distributed in humans and consumption animals (Supplementary Table 1). Combined with the antimicrobial susceptibility testing, we can deduce that the *E. coli* CAU16175 isolate has MDR and is difficult to control.

The *E. coli* CAU16175 isolate also carries the *mcr-1.1* gene in pCAU16175_3 (IncFII type). The emergence of the *mcr-1* gene can be traced back to the *E. coli* isolated in the 1980s, and the outbreak of chicken-derived *mcr-1*–containing *E. coli* started in 2009 (46).The *mcr-1* gene has been characterized in various genetic backgrounds and observed on a variety of plasmid type, including Incl2, IncX4, IncHI2, IncP, IncHI1, IncFII, IncFI, IncFIB, F18:A-:B+, IncY, IncK, and phage-like plasmid (29). Most of the recently reported the *mcr-1* genes were primarily mobilized by an IS*Apl1* composite Tns Tn*6330* and Tn*6390* (47, 48). The IS*Apl1* would be lost over time, leading to the stability of the *mcr-1* gene on plasmids (18, 49). Thus, only the truncated IS*Apl1* will lose the ability to transfer the *mcr-1* gene.

Furthermore, the *mcr-1.1* gene is located within the Tn*2* derivative in pCAU16175-3. From the Tn registry website (https://transposon.lstmed.ac.uk/), we noticed the *mcr-5* gene and the *mcr-3.6* gene could be mobilized by Tn*6452* and Tn*6518* (belonged to Tn*2* family). For the Tn*6452*, the *mcr-5* gene was embedded within a Tn*3*-family Tn with 38-bp inverted repeats and flanked by 5-bp DRs, which were usually generated during the insertion (50, 51). Although DRs appear at the flanking site of the Tn*2* derivative, the whole structure did not move by transposition because of truncated *tnpA* and deleted *tnpR*, which are essential to occur transposition for Tn*2*.

## Conclusions

The current work shows the genetic characteristics of five DEC strains that exhibited MDR, including colistin resistance. Our data indicate that the ST4214 ETEC/STEC carried MDR and multivirulence genes and that the *E. coli* CAU16175 contains IS*26*-enriched MDR region and the *mcr-1.1* gene, which is located within a Tn*2* derivative. The coexistence of MDR and multivirulence in DEC may seriously compromise the effectiveness of clinical therapy, and heightened efforts are needed to control their dissemination.

## Data Availability Statement

The datasets presented in this study can be found in online repositories. The names of the repository/repositories and accession number(s) can be found below: https://www.ncbi.nlm.nih.gov/, SRR10813965; https://www.ncbi.nlm.nih.gov/, SRR10828049; https://www.ncbi.nlm.nih.gov/, SRR10828048; https://www.ncbi.nlm.nih.gov/, SRR10828047; and https://www.ncbi.nlm.nih.gov/, SRR10828046.

## Ethics Statement

The animal study was reviewed and approved by the Animal Ethics Committee of the China Agricultural University under the protocol CAU20140616-1.

## Author Contributions

LG and YZ conceived and designed the experiments. LG, JW, SW, JS, and XW performed the experiments. LG analyzed the sequencing data and wrote the manuscript. YZ revised the manuscript. All authors contributed to the article and approved the submitted version.

## Conflict of Interest

The authors declare that the research was conducted in the absence of any commercial or financial relationships that could be construed as a potential conflict of interest.

## Abbreviations

DEC: diarrheagenic *Escherichia coli*
WGS: whole-genome sequencing
MDR: multidrug resistance
ETEC: enterotoxigenic *E. coli*
STEC: Shiga-toxigenic *E. coli*
aEPEC: atypical enteropathogenic *E. coli*
tEPEC: typical enteropathogenic *E. coli*
PWD: postweaning diarrhea
IS: insertion sequence
Tn: transposon
PCR: polymerase chain reaction
DR: direct repeat.
