# Supplementary material for: Genome Characterization of mcr-1–Positive Escherichia coli Isolated From Pigs With Postweaning Diarrhea in China
Source: Front Vet Sci. 2020 Aug 25;7:503. doi: 10.3389/fvets.2020.00503 (PMC7479848; doi:10.3389/fvets.2020.00503)
Supplement: Supplementary file 1 [file Data_Sheet_1.docx]

Supplementary Material

# Supplementary Tables

# Supplementary Table 1. Information of 30 IncHI2 type plasmids showing homologous plasmid backbone structure (more than 90% coverage and 99% identity compared with pCAU16175_1).

| Plasmid ID | Accession Number | Isolation Source | Plasmid Size (bp) | Submission Date | Species | Region | Coverage (%) | Identity  (%) |
| --- | --- | --- | --- | --- | --- | --- | --- | --- |
| pSH16G4498 | MH522423 | Homo sapiens | 297846 | 2019 | *Salmonella Typhimurium* | China | 99 | 99.78 |
| pSH16G2457 | MH522421 | Homo sapiens | 299616 | 2019 | *Salmonella Typhimurium* | China | 99 | 99.75 |
| pHNYJC8 | KY019259 | Chicken meat | 249153 | 2016 | *Escherichia coli* | China | 97 | 99.80 |
| pHNLDF400 | KY019258 | Patient | 249152 | 2016 | *Escherichia coli* | China | 97 | 99.80 |
| pHXY0908 | KM877269 | Chicken stool | 249144 | 2014 | *Salmonella Typhimurium* | China | 97 | 99.76 |
| p1 | LT795115 |  | 246444 | 2016 | *Salmonella Typhimurium* | United Kingdom | 96 | 99.80 |
| pHK0653 | KT334335 | Homo sapiens stool | 244851 | 2015 | *Salmonella Typhimurium* | Hong Kong | 95 | 99.80 |
| p12519A | CP041174 | Homo sapiens feces | 248746 | 2019 | *Salmonella Enteritidis* | China | 95 | 99.79 |
| pHNSHP45-2 | KU341381 | Pig feces | 251493 | 2015 | *Escherichia coli* | China | 95 | 99.75 |
| pMCR-H8 | CP029215 | Homo sapiens feces | 203941 | 2018 | *Escherichia coli* | China | 95 | 99.74 |
| pLSB54-*mcr-1* | MG773376 |  | 251657 | 2018 | *Escherichia coli* | China | 95 | 99.74 |
| pP2-3T | MG014722 |  | 392275 | 2017 | *Escherichia coli* | China | 94 | 99.78 |
| pSH16G0648 | MH522418 | Patient | 244206 | 2018 | *Salmonella Typhimurium* | China | 94 | 99.74 |
| pA3T | KX421096 | Chicken feces | 253092 | 2016 | *Salmonella Indiana* | Hong Kong | 92 | 99.80 |
| pC629 | CP015725 | Chicken carcass | 210106 | 2016 | *Salmonella enterica* | China | 92 | 99.79 |
| pSH16G2456 | MK477616 | Homo sapiens | 251053 | 2019 | *Salmonella enterica* | China | 92 | 99.74 |
| pSH15G1450 | MK477606 | Homo sapiens | 254389 | 2019 | *Salmonella enterica* | China | 92 | 99.74 |
| pSH16G4918 | MK477619 | Homo sapiens | 217630 | 2019 | *Salmonella enterica* | China | 92 | 99.74 |
| pSH15G1428 | MK477605 | Homo sapiens | 252852 | 2019 | *Salmonella enterica* | China | 92 | 99.74 |
| pSH16G4466 | MK477617 | Homo sapiens | 249071 | 2019 | *Salmonella enterica* | China | 92 | 99.74 |
| p87912 | CP041180 | Chicken | 236217 | 2019 | *Salmonella Indiana* | China | 92 | 99.70 |
| pSH16G1394 | MK477614 | Homo sapiens | 251867 | 2019 | *Salmonella enterica* | China | 91 | 99.80 |
| pSJ_255 | CP011062 | Pheasant duodenum | 255368 | 2015 | *Escherichia coli* | China | 91 | 99.72 |
| pSH16G4511 | MK477618 | Homo sapiens | 219141 | 2019 | *Salmonella enterica* | China | 91 | 99.74 |
| pECJS-59-244 | KX084394 | Piglet feces | 243572 | 2017 | *Escherichia coli* | China | 91 | 99.66 |
| pTJWQ005 | CP040457 | Homo sapiens | 254752 | 2019 | *Salmonella enterica* | China | 90 | 99.79 |
| pSH16G4525 | MH522424 | Homo sapiens | 278996 | 2018 | *Salmonella enterica* | China | 90 | 99.78 |
| pSH15G1598 | MK477609 | Homo sapiens | 176207 | 2019 | *Salmonella enterica* | China | 90 | 99.86 |
| pSH15G1571 | MK477608 | Homo sapiens | 175206 | 2019 | *Salmonella enterica* | China | 90 | 99.86 |
| pD90-1 | CP022451 | Chicken | 222470 | 2017 | *Salmonella enterica* | China | 90 | 99.68 |

# Supplementary Table 2. Information of 32 IncFII type plasmids showing homologous plasmid backbone structure (more than 80% coverage and 94% identity compared with pCAU16175_3) and plasmid-*mcr-1* pKP81-BE.

| Plasmid ID | Accession Number | Isolation Source | Plasmid Size (bp) | Submission Date | Species | Region | Coverage (%) | Identity  (%) |
| --- | --- | --- | --- | --- | --- | --- | --- | --- |
| pEC1515-3 | CP021847 | Homo sapiens | 84091 | 2017 | *Escherichia coli* | Taiwan | 89 | 97.09 |
| pEC974-3 | CP021843 | Homo sapiens | 78672 | 2017 | *Escherichia coli* | Taiwan | 99 | 97.68 |
| pH1038-142 | KJ484634 | Homo sapiens | 142875 | 2016 | *Escherichia coli* | Ireland | 87 | 97.17 |
| pFORC_081_1 | CP029058 | Chicken | 253947 | 2019 | *Escherichia coli* | South Korea | 86 | 97.25 |
| plasmid R1 | KY749247 | Chicken stool | 97566 | 2017 | *Salmonella enterica* | USA | 86 | 96.83 |
| ATCC 43896 plasmid unnamed3 | CP024281 | Infant diarrheic stool | 84894 | 2017 | *Escherichia coli* | USA | 85 | 96.68 |
| plasmid 39R861-3 | MK092064 |  | 61297 | 2019 | *Escherichia coli* | Australia | 83 | 97.13 |
| plasmid: RCS28_pI | LT985227 |  | 95442 | 2018 | *Escherichia coli* | France | 83 | 96.76 |
| pM110_FII | AP018140 |  | 81783 | 2019 | *Escherichia coli* | Myanmar | 82 | 97.88 |
| p14408_1 | LT599826 |  | 137186 | 2017 | *Escherichia coli* | Germany | 82 | 96.83 |
| pSB4816 | MF363048 | Horse genital tract | 78083 | 2018 | *Klebsiella pneumoniae* | Australia | 82 | 96.70 |
| RCS84_p | LT985305 |  | 83309 | 2018 | *Escherichia coli* | France | 82 | 96.69 |
| pZM3 | MK797990 |  | 166767 | 2019 | *Salmonella enterica* | Australia | 82 | 94.46 |
| pCTXM-2271 | MF589339 |  | 222492 | 2017 | *Escherichia coli* | China | 81 | 97.24 |
| pHeBE7 | KT002541 | Chicken | 86015 | 2015 | *Escherichia coli* | China | 81 | 96.86 |
| pMC-NDM | HG003695 |  | 87619 | 2016 | *Escherichia coli* | Poland | 81 | 96.86 |
| pM109_FII | AP018139 |  | 90294 | 2019 | *Escherichia coli* | Myanmar | 81 | 96.86 |
| pA74T | MG014720 | Duck | 322966 | 2019 | *Escherichia coli* | China | 81 | 96.84 |
| 2009C-3133 plasmid unnamed2 | CP013026 | Homo sapiens | 63800 | 2015 | *Escherichia coli* | USA | 81 | 96.71 |
| 165 plasmid unnamed1 | CP020510 | Homo sapiens | 118182 | 2018 | *Escherichia coli* | USA | 81 | 97.16 |
| pSCKLB138-1 | MH161192 |  | 117559 | 2018 | *Klebsiella pneumoniae* | China | 81 | 97.03 |
| pL65-2 | CP034739 |  | 145346 | 2019 | *Escherichia coli* | China | 80 | 97.25 |
| pP2-3T | MG014722 | Pig | 392275 | 2018 | *Escherichia coli* | China | 80 | 97.24 |
| MS14386 plasmid 3 | LR130554 |  | 93927 | 2019 | *Escherichia coli* | Australia | 80 | 97.27 |
| M19 plasmid B | CP010223 | Mouse | 61063 | 2016 | *Escherichia coli* | China | 80 | 96.69 |
| M15 plasmid B | CP010215 | Mouse | 61058 | 2016 | *Escherichia coli* | China | 80 | 96.69 |
| M11 plasmid C | CP010209 | Mouse | 61005 | 2016 | *Escherichia coli* | China | 80 | 96.69 |
| pCHL5009T-102k-mcr3 | CP032937 | Homo sapiens | 101860 | 2018 | *Escherichia coli* | New Zealand | 80 | 96.86 |
| pHNHN21 | KX246267 | Pigeon | 83435 | 2017 | *Escherichia coli* | China | 80 | 96.86 |
| p4540-2 | CP041534 | Homo sapiens | 88800 | 2019 | *Escherichia coli* | USA | 80 | 95.78 |
| pEC129_2 | CP038455 | Homo sapiens | 117599 | 2019 | *Escherichia coli* | Japan | 80 | 97.17 |
| pRmtB1_005237 | CP026579 | Homo sapiens | 100229 | 2019 | *Escherichia coli* | China | 80 | 97.17 |
| pKP81-BE | KU994859 | Pig | 91041 | 2016 | *Escherichia coli* | Belgian | 13 | 94.38 |

# Supplementary Table 3. The primers used in this study.

| Primer ID | Direction | Sequence (5'→3') | Product size (bp) |
| --- | --- | --- | --- |
| P1 | F | TGTATGTCGGTGTATTTGGGGTCTG | 2335 |
|  | R | CAAAGTCAGCACCAGATACGCAAGC |  |
| P2 | F | TGCGACCATTTGCCGTGCTTATCAT | 3557 |
|  | R | ATTCGGTAAAGTTCGTCACACACAT |  |
| P3 | F | TACAACGAGGACGGATAATACGCTT | 4851 |
|  | R | CAAAAAGTTTCGTCATAAATGGCTC |  |
| P4 | F | GCAACCAAGCCTGATATGCG | 521 |
|  | R | CGCTTAAAATACGCAGGCCC |  |
| P5 | F | TTGCTGGTTGCGACCATTTG | 531 |
|  | R | TGTCAAACCAAGTGCAACGA |  |
| P6 | F | TCCTTGAGAGTTTTCGCCCC | 634 |
|  | R | TGACTCCCCGTCGTGTAGAT |  |
| P7 | F | TCATGCAGCTCCACCGATTT | 626 |
|  | R | CTGTTCAGCTACTGACGGGG |  |
| P8 | F | TCAACACATCGAGGTCCGTC | 737 |
|  | R | TCGCAACCATAGCGTATCCC |  |
| P9 | F | CTGCATACCGGTTTCTGGGT | 349 |
|  | R | CTGCTTACCAGGCGCATTTC |  |
| P10 | F | CTGCAGGATAGTCTGAGGCG | 942 |
|  | R | AAACTGGTATCCCAGCAGCC |  |

# P1, amplified for nucleotide location 2062-4396 in pCAU16175_3; P2, amplified for nucleotide location 4282-7838 in pCAU16175_3; P3, nucleotide location 7441-12291 in pCAU16175_3; P4-P9, amplified for *mcr-1*, *pap2*, *bla*_TEM-1B_, IS*1X2*, *tet*(M), IS*15DI* and *tnpA*, respectively.

# Supplementary Figure

**
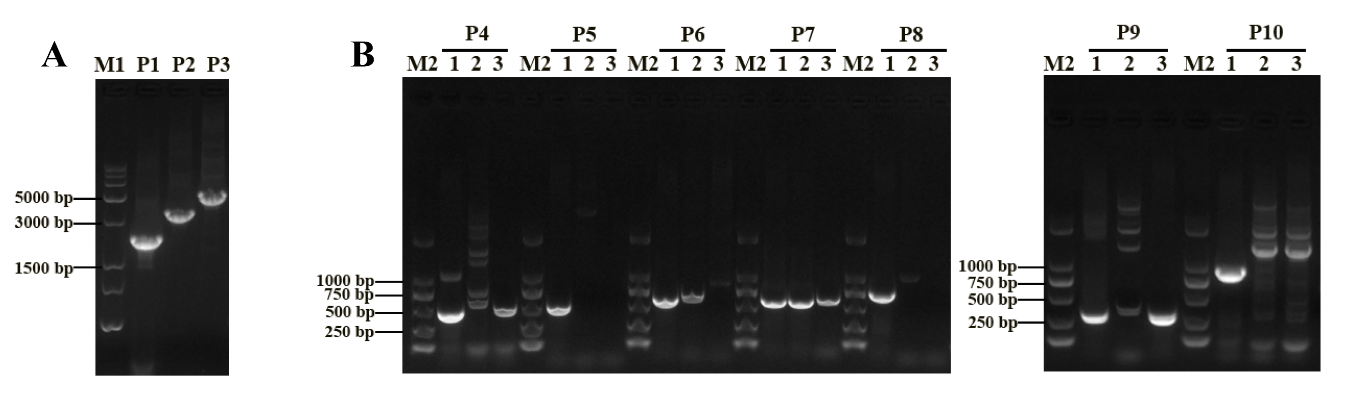
**

**Supplementary Figure 1.** Confirmation of the *mcr-1* gene located within a destructed Tn*2* transposon and transferred to the receptor J53 by PCR. (**A**) M1, 15K DNA marker. P1, 2335 bp (nucleotide location 2062-4396 in pCAU16175_3) PCR products generated by primer 1. P2, 3557 bp (nucleotide location 4282-7838 in pCAU16175_3) PCR products generated by primer 2. P3, 4851 bp (nucleotide location 7441-12291 in pCAU16175_3) PCR products generated by primer 3. (**B**) M2, DL2000 DNA marker. 1, CAU16175. 2, J53. 3, J53-*mcr-1*. The PCR products generated by primer 4-10 amplified for *mcr-1*, *pap2*, *bla*_TEM-1B_, IS*1X2*, *tet*(M), IS*15DI* and *tnpA* depicted on the straight line, respectively. A series of PCR primers is listed in Table S3.
